# Effects of Drug-Coated Balloon Therapy on CT Imaging Results and Levels of Vascular Inflammatory Cytokines in Patients with Arteriosclerosis Obliterans Lesions

**DOI:** 10.1155/2022/4793547

**Published:** 2022-09-22

**Authors:** Yanlin Yang

**Affiliations:** Department of Interventional Radiology, Harrison International Peace Hospital, Hengshui, Hebei 053000, China

## Abstract

**Objective:**

The aim of the study is to explore the effects of drug-coated balloon (DCB) therapy on computed tomography (CT) imaging results and levels of vascular inflammatory cytokines in patients with arteriosclerosis obliterans (ASO) lesions.

**Methods:**

A total of 200 patients with ASO lesions admitted to our hospital from January 2021 to December 2021 were enrolled. According to the random number table method, they were divided into observation groups (*n* = 100) and control groups (*n* = 100). The observation group was treated with DCB, while the control group was treated with ordinary balloon. At 1 month after surgery, the clinical curative effect was evaluated by ankle-brachial index (ABI). The image quality was compared and vascular patency was evaluated by arterial ultrasound and CT angiography. The minimum luminal diameter (MLD) and late luminal loss (LLL) were recorded. Before and at 1 month after surgery, the severity of disease was assessed by Rutherford grading of lower limb ischemia. Before, at 7 d and 1 month after surgery, inflammatory factors [serum matrix metalloproteinase-9 (MMP-9), monocyte chemoattractant protein-1 (MCP-1), high sensitivity C-reactive protein (hs-CRP), interleukin-4 (IL-4), interleukin-6 (IL-6)] were compared between the two groups. The occurrence of postoperative complications was recorded.

**Results:**

The clinical response rate in the observation group was significantly higher than that in the control group (97.00% *vs* 89.00%) (*P* < 0.05). The restenosis rate in the observation group was significantly lower than that in the control group (1.00% *vs* 7.00%) (*P* < 0.05). The MLD in the observation group was significantly longer than that in the control group, and the LLL was significantly lower than that in the control group (*P* < 0.05). There was no significant difference in image quality between the two groups (*P* > 0.05). After surgery, disease severity in both groups was relieved, which was milder in the observation group than in the control group (*P* < 0.05). At 7 d and 1 month after surgery, levels of MMP-9, MCP-1, hs-CRP, IL-4, and IL-6 in both groups were decreased, which were lower in the observation group than in the control group (*P* < 0.05). There was no significant difference in the incidence of complications between the two groups (6.00% *vs* 7.00%) (*P* > 0.05).

**Conclusion:**

The curative effect of DCB is good on patients with ASO lesions, which can reduce the restenosis rate, control luminal loss, relieve inflammatory response, and improve disease severity.

## 1. Introduction

Arteriosclerosis obliterans (ASO) refers to arteriosclerosis in the lower body. Common symptoms include lower extremity pain, chills, and inability to move. If the disease cannot be treated in time, the symptoms of lower extremity ulceration will also occur. In severe cases, it may lead to amputation, which is harmful to the patient's physical and mental health [1]. In the past, an open surgical bypass was used for treatment, but due to the large trauma and risk of surgery, coupled with the influence of anesthesia and other factors, the patient's inflammatory factors changed greatly and the prognosis was poor [2]. A large number of released inflammatory factors cause the body to show a high inflammatory state, which will aggravate the damage to vascular function and more easily lead to the occurrence of postoperative restenosis in patients. Therefore, it is of great significance for ASO patients to take effective measures to reduce the inflammatory reaction caused by surgical trauma. In recent years, minimally invasive treatment represented by endovascular interventional therapy has become the dominant method for ASO. Drug-coated balloons (DCB) have become a new clinical treatment trend because of their minimally invasive, repeatable, limb preservation, and other characteristics [3]. Studies have shown [4] that DCB has a significant advantage in reducing postoperative restenosis rates in patients with ASO due to its surface covering with antiangiogenic drugs. However, there are few reports on the effect of this procedure on inflammatory factors in patients with ASO. Therefore, this study explored the effect of DCB treatment on CT imaging results and levels of vascular inflammatory cytokines in patients with ASO, in order to guide clinical medication and improve postoperative vascular flow rate.

## 2. Materials and Methods

### 2.1. General Information

A total of 200 patients with ASO lesions who were admitted to our hospital from January 2021 to December 2021 were selected. The inclusion criteria were as follows: ① meet the relevant diagnostic criteria of ASO [5]; ② have surgical indications; and ③ patients and their family members understand and agree to this study. The exclusion criteria were as follows: ① severe hepatic and renal insufficiency (hepatic function grade C, renal function stage 4, or above); ② severe cardiac insufficiency (cardiac function grade 3 or above); ③ iodine allergy; ④ the patient's condition deteriorated rapidly in the preoperative preparation stage; and ⑤ no contraindication to surgery. The enrolled patients were divided into an observation group (*n* = 100) and control group (*n* = 100) by the 1 : 1 random number table method. In the observation group, there were 54 males and 46 females aged 52–74 years, with an average of (63.27 ± 4.64) years; 57 cases of hypertension, 36 cases of diabetes mellitus, and 14 cases of coronary heart disease; the lesion length was 10–17 cm, with an average of (13.57 ± 1.26) cm. In the control group, there were 61 males and 39 females, aged 52–74 years, with an average of (63.32 ± 4.43) years; 51 cases of hypertension, 38 cases of diabetes mellitus, and 19 cases of coronary heart disease; the lesion length was 10–17 cm, with an average of (13.48 ± 1.30) cm. There was no significant difference in the general data between the two groups (*P* > 0.05).

### 2.2. Methods

Surgical method: The patient is placed in the supine position, after routine draping and disinfection, local anesthesia is applied, and then puncture is performed (anterograde ipsilateral femoral artery or retrograde lateral femoral artery). A 4-F vascular sheath was placed, heparin sodium was injected intravenously, heparin was added according to the operation time, and angiography was performed to understand the lesion location and the location of the patient. If there are stenotic and occluded arteries in all limbs, conventional catheter and guide wire techniques should be used to pass through the stenotic lesions, and SIA technology should be used to pre-dilate the occlusive lesions with a balloon with a smaller diameter. Then, we use balloon dilatation. The observation group was expanded with DCB (surface-coated paclitaxel, Beijing Kangtai Huizhong Technology Co., Ltd.) with the same diameter as the original stent inner diameter for 2–3 minutes (10–12 atmospheres); the control group was expanded with ordinary balloons (10–12 atmospheres). If the residual stenosis after expansion is ≥ 30% or there is a dissection that affects blood flow, a stent is implanted.

Postoperative intervention: we routinely subcutaneously inject low molecular weight heparin sodium (manufacturer: Italian Alfa Wassermann SpA, approval number: registration number H20140282) 0.1 mL/kg, 2 times/d; alprostadil injection (manufacturer: Harbin Pharmaceutical Group Bioengineering Co., Ltd., approval number: Guoyao Zhunzi H20084565) intravenous drip, 10 *μ*g/time, 2 times/d, continuous medication for 3 days. After discharge from the hospital, we continue to use dual antibodies (aspirin and clopidogrel) for more than 6 months.

Examination method: Philips 256-slice spiral CT was used, the patient was in the supine position, and the scanning range: 2 cm above the bifurcation of the abdominal aorta to the toes of both lower extremities. The setting parameters are 120 kw, 180 mA, the layer thickness is 10 mm, and the building layer thickness is 10 mm. After the scan, the data were transferred to the GE 4.0 workstation for multislice reconstruction, maximum density projection, and volume reconstruction, and then combined with plain scan and enhanced axial images for analysis.

### 2.3. Observation Indicator

#### 2.3.1. Clinical Efficacy and Imaging Indicators

One month after the operation, an ankle-brachial index (ABI) examination [6] was used to evaluate the clinical efficacy. The calculation method of ABI: The systolic blood pressure ratio of the posterior tibial artery to the brachial artery was measured once bilaterally and the mean value was taken (normal values range from 0.9 to 1.3). Effective: ABI increased by more than 0.2, the ASO site was safely opened or residual stenosis was less than 30%, distal arterial pulsation resumed, and clinical symptoms and signs improved or disappeared. Effective rate = Effective cases/total cases × 100%. Lower extremity arterial Doppler ultrasonography was used to measure and calculate the restenosis rate (the stenosis rate ≥50% is the vascular restenosis [4]) and record the minimum luminal diameter (MLD) and target vessel late luminal loss (LLL).

#### 2.3.2. Image Quality

CT examination was performed 1 month after the operation, and the image quality was analyzed by two or more senior radiologists [7]. Level 1: artery are clearly visible without artifact. Level 2: arteries are clearly displayed, and peripheral veins can be slightly displayed. Level 3: arteries can be clearly displayed, but peripheral veins are clearly visualized. Level 4: arterial vessels are not visualized. The higher the grade, the worse the CT contrast image quality.

#### 2.3.3. Severity of Illness

At 1 day before surgery and 1 month after surgery, the Rutherford classification of lower extremity ischemia [8] was used to evaluate the severity of the patient's condition. Level 0: Asymptomatic. Level 1: Mild intermittent claudication, treadmill test can be completed. Level 2: Moderate intermittent claudication. Level 3: Severe intermittent claudication, unable to complete the treadmill test. Level 4: Pain at rest may occur due to replacement of the position above the foot. Level 5: Slight tissue defect. Level 6: Tissue ulceration, gangrene. The lower the grade, the better the patient's condition is controlled.

#### 2.3.4. Inflammatory Factors

At 1 day before surgery and 7 days and 1 month after surgery, 3–4 ml of fasting blood was drawn from the patient with a vacuum blood collection tube containing a coagulant on an empty stomach in the morning, and the samples were stored in a refrigerator at −20°C Matrix metalloproteinase -9 (MMP-9), monocyte-macrophage chemoattractant factor -1 (MCP-1), and high-sensitivity C-reactive protein (hs-CRP) were all detected by enzyme-linked immunosorbent assay (Reagents and kits were provided by Shanghai Yaji Biotechnology Co., Ltd.), and serum interleukin-4 (IL-4) and interleukin-6 (IL-6) levels were detected by chemiluminescence detection (reagents and kits were provided by Beijing Soleibo Technology Co., Ltd.); all tests were performed by the same physician in strict accordance with the test instructions.

#### 2.3.5. Complications

All patients were followed up for one month after the operation. Postoperative complications such as infection, puncture site hematoma, renal function impairment, and thrombosis were recorded in the two groups of patients.

### 2.4. Statistical Processing

The clinical data of patients with ASO lesions in this study were analyzed by SPSS 22.0 software, and the measurement data that met normal distribution and homogeneity of variance, such as MLD, LLL, and inflammatory factor indicators, were expressed as (mean ± sd). The differences between the observation group and the control group without time points were compared using a two-sample independent *t* test, and the differences between groups with time points were compared with repeated measures analysis of variance. Before and after the operation, the differences between the observation group and the control group were compared by paired *t* test, the count data were expressed by case (%), the *χ*^2^ test was performed, and the rank data were subjected to the rank sum test, suggesting statistical significance.

## 3. Results

### 3.1. Comparison of Clinical Efficacy and Vascular Patency between the Two Groups

The clinical efficacy rate of patients in the observation group was 97.00%, which was significantly higher than that in the control group (89.00%) (*P* < 0.05); the restenosis rate was 1.00%, which was significantly lower than 7.00% in the control group (*P* < 0.05) as shown in Table 1.

### 3.2. Comparison of Imaging Indexes between the Two Groups

**Figure 1:**
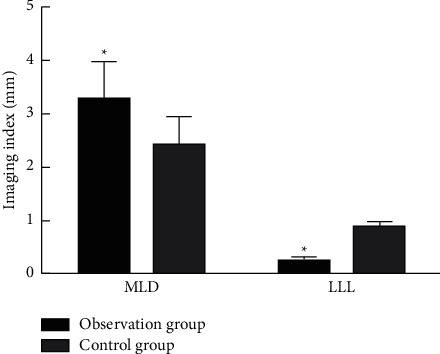
Comparison of imaging indicators between the two groups. Compared with the control group, ^*∗*^*P* < 0.05.

The MLD in the observation group was significantly higher than that in the control group and the LLL was significantly lower than that in the control group (*P* < 0.05), as shown in Figure 1.

### 3.3. Comparison of Image Quality between the Two Groups

There was no significant difference in image quality between the two groups (*P* > 0.05). See Table 2 for details.

### 3.4. Comparison of Disease Severity between the Two Groups

Before surgery, there was no significant difference in the severity of illness between the two groups (*P* > 0.05); after the operation, the severity of patients in both groups decreased, and the observation group was lower than the control group (*P* < 0.05). See Table 3 for details.

### 3.5. Comparison of Inflammatory Factor Levels between the Two Groups

**Figure 2:**
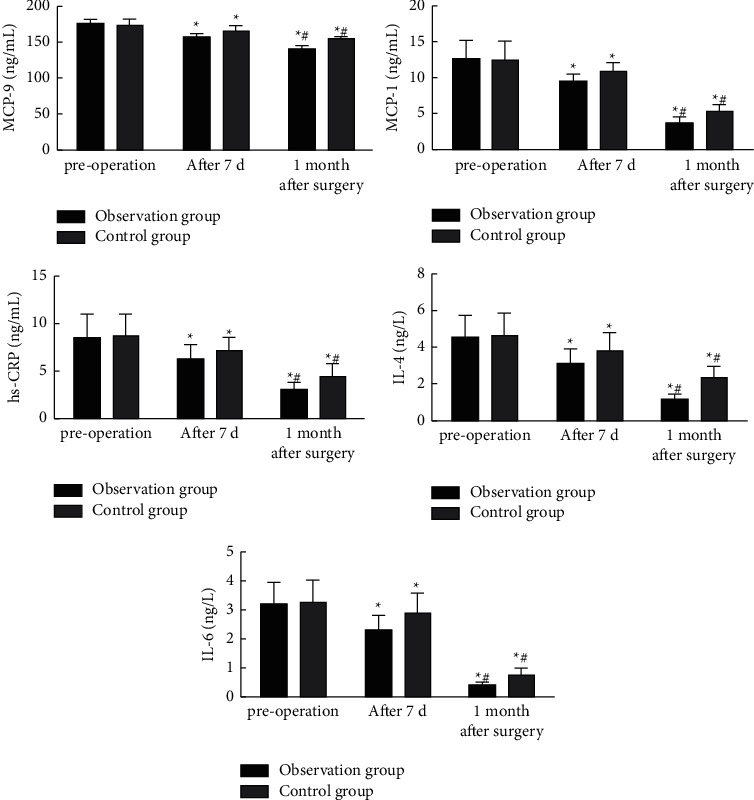
Comparison of inflammatory factor levels between the two groups. Compared with preoperative, ^*∗*^*P* < 0.05; Compared with postoperative 7d, ^#^*P* < 0.05.

Before the operation, there was no significant difference in MMP-9, MCP-1, hs-CRP, IL-4, and IL-6 between the two groups (*P* > 0.05). After the operation, the levels of MMP-9, MCP-1, hs-CRP, IL-4 and IL-6 in two groups were decreased as compared with those before the operation; the decrease was more significant 1 month after the operation as compared with that 7 days after the operation; and the observation group was lower than the control group (*P* < 0.05). See Figure 2 for details.

### 3.6. Comparison of the Incidence of Complications between the Two Groups

At follow-up within 1 month after the operation, there was no significant difference in the incidence of complications between the two groups (6.00% vs 7.00%) (*P* > 0.05). See Table 4 for details.

## 4. Discussion

In recent years, with the increase of risk factors such as hypertension and diabetes, the incidence of ASO lesions has also increased significantly, mainly manifested as a series of symptoms and signs such as intermittent claudication, weakened or disappeared arterial pulse, and nutritional supply disorder of limb tissue. At present, there are many clinical treatment methods for patients with ASO lesions, including drug therapy, balloon dilation, and stent implantation. However, studies have pointed out [9] that such treatment methods are not effective. Therefore, the search for safer and more effective treatment methods has become a focus of clinical attention. This article will discuss the application value of DCB therapy in patients with ASO lesions from the aspects of imaging indicators, efficacy, inflammatory factors, severity, and complications, in order to provide an evidence-based basis for the selection of clinical treatment methods.

The clinical efficacy of the observation group was significantly higher than that of the control group, and the restenosis rate and disease severity were significantly lower than those of the control group, which was consistent with previous research results [10], suggesting that DCB has a higher clinical efficacy in the treatment of patients with ASO lesions and can improve the patient's condition and reduce postoperative restenosis rate. Analysis of the reasons may be because DCB is coated with paclitaxel, which can inhibit intimal hyperplasia, on the surface of the traditional balloon, which can not only play a role in expanding the lesion but also release paclitaxel into the local vascular intima, so that the paclitaxel can interact with the lesion and the blood vessel wall is fully contacted, and the drug penetrates into the arterial wall through antiproliferation and anti-inflammatory effects, inhibiting and delaying the migration and proliferation of smooth muscle cells, thereby achieving the purpose of continuously inhibiting the mitosis of smooth muscle cells and inhibiting intimal hyperplasia [11, 12]. So, DCB can increase the long-term patency rate of blood vessels, reduce the restenosis rate, effectively improve the patient's condition, relieve clinical symptoms, and improve the curative effect. In this study, the MLD of the observation group was significantly higher than that of the control group, and the LLL was significantly lower than that of the control group, indicating that DCB treatment is beneficial to control lumen loss and effectively maintain adequate vascular lumen in patients. Because DCB combines the balloon with the drug elution technology, the balloon shows that the drug paclitaxel, which inhibits cell proliferation, is attached to the balloon, which hinders the promotion of smooth muscle cells to a certain extent, and can ensure sufficient lumen diameter and avoid lumen loss.

The imbalance of vascular endothelial cell function leads to an increase in the levels of inflammatory mediators and cytokines secreted and released by the vascular endothelial cells, resulting in a long-term microinflammatory response in the blood vessels. The most important part of atherosclerosis is chronic inflammation, which is significantly related to the pathological changes of vascular disease. Therefore, the imbalance of vascular endothelial cell function leads to a long-term microinflammatory reaction in blood vessels, which is the pathological basis of the ASO. MMP-9 is an important factor promoting the development of atherosclerosis, and its level reflects the formation of atherosclerosis and plaque stability; IL-4 and IL-6 act as proinflammatory factors, and their elevated levels reflect the progression of atherosclerosis to a certain extent; hs-CRP is a nonspecific inflammatory factor secreted by the liver, and studies have pointed out [13] that its elevated level is related to the occurrence, development, and prognosis of acute coronary syndrome, and is an important inflammatory factor in the development of atherosclerosis. As an initiating factor and marker of inflammation, MCP-1 has chemotaxis and activation effects on monocytes, which can expand the inflammatory response and participate in the progression of atherosclerosis [14]. In this study, after seven days and one month of treatment, the levels of MMP-9, MCP-1, hs-CRP, IL-4, and IL-6 in the observation group were significantly lower than those in the control group, indicating that DCB treatment for patients with ASO lesions was conducive to reducing inflammatory factors, which might be related to the anti-cell proliferation and inhibition of vascular endothelial injury by paclitaxel, a DCB surface coating drug.

In conclusion, DCB has good safety and efficacy in the treatment of patients with ASO lesions, which is helpful to control the lumen loss of patients, maintain the vascular lumen, reduce the restenosis rate and the level of inflammatory factors, and achieve the purpose of improving the disease. However, the sample size included in this study was relatively small and it lacked long-term efficacy and comprehensiveness. Therefore, the specific clinical benefit should be confirmed by expanding the sample size.

## Figures and Tables

**Table 1:** Comparison of clinical efficacy and vascular patency between the two groups (*n* (%)).

Groups	Effective rate	Vascular restenosis rate
Observation group (*n* = 100)	97 (97.00)	1 (1.00)
Control group (*n* = 100)	89 (89.00)	7 (7.00)
*χ* ^2^	4.916	4.688
*P*	0.027	0.030

**Table 2:** Comparison of image quality between the two groups (n (%)).

Groups	Level 1	Level 2	Level 3	Level 4
Observation group (*n* = 100)	86 (86.00)	12 (12.00)	2 (2.00)	0 (0.00)
Control group (*n* = 100)	81 (81.00)	11 (11.00)	5 (5.00)	3 (3.00)
*Z*	1.210			
*P*	0.272			

**Table 3:** Comparison of disease severity between the two groups (*n* (%)).

Groups	*Preoperation*						*Postoperation*					
	Level 1	Level 2	Level 3	Level 4	Level 5	Level 6	Level 1	Level 2	Level 3	Level 4	Level 5	Level 6
Observation group (*n* = 100)	5 (5.00)	34 (34.00)	27 (27.00)	21 (21.00)	13 (13.00)	0 (0.00)	37 (37.00)	31 (31.00)	26 (26.00)	5 (5.00)	1 (1.00)	0 (0.00)
Control group (*n* = 100)	7 (7.00)	29 (29.00)	33 (33.00)	21 (21.00)	10 (10.00)	0 (0.00)	29 (29.00)	27 (27.00)	29 (29.00)	9 (9.00)	6 (6.00)	0 (0.00)
*Z*	0.030						3.870					
*P*	0.858						0.049					

**Table 4:** Comparison of the incidence of complications between the two groups (*n* (%)).

Groups	Postoperative infection	Hematoma at puncture site	Impaired renal function	Thrombogenesis	Complication rate
Observation group (*n* = 100)	2 (2.00)	1 (1.00)	1 (1.00)	2 (2.00)	6 (6.00)
Control group (*n* = 100)	3 (3.00)	2 (2.00)	3 (3.00)	1 (1.00)	9 (9.00)
*χ* ^2^					0.649
*P*					0.421

## Data Availability

The data used and/or analyzed during the current study are available from the corresponding author upon request.
